# Investigation of patients’ access to EHR data via smart apps in Chinese Hospitals

**DOI:** 10.1186/s12911-021-01425-w

**Published:** 2021-07-30

**Authors:** Ting Shu, Fan Xu, Hongxia Li, Wei Zhao

**Affiliations:** 1 Department of Health Care IT, National Institute of Hospital Administration, NHC, Building 3, Yard 6, Shouti South Road, Haidian District, Beijing, 100044 China; 2 Department of Information Center, Fuwai Hospital, National Center for Cardiovascular Diseases, Chinese Academy of Medical Sciences and Peking Union Medical College, No.167 North Lishi Road, Xicheng District, Beijing, 100037 China

**Keywords:** EHR data release, Electronic health records, Smart apps, Cost saving, Information security protection

## Abstract

**Background:**

Given that China has encouraged EHR usage in hospitals for more than a decade, patients’ access to their own EHR data is still not as widely utilized as expected.

**Methods:**

We cultivated a survey with four categories and field interviews of measures to identify whether hospitals have already released EHR data to patients, inpatients or outpatients, the top EHR release contents and the most popular release software.

**Results:**

Of the 1344 responding hospitals from 30 provinces nationwide, 41.37% of hospitals have already released their EHR data to patients, of which 97.12% are through smart apps. More than 91% of hospitals use WeChat, and 32.37% of hospitals developed their own standalone apps or use vendors’ apps. A total of 54.63% were released to both outpatients and inpatients, and the top release contents were all objective. A rough estimation is made that releasing EHR data to patients via smart apps may save the hospital 15.9 million RMB per year and patients 9.4 million RMB altogether.

**Conclusions:**

EHR data release is believed to bring both patient and hospital cost savings and efficiency gains but is still considered spontaneous and requires legal support and government regulation.

## Background

While electronic health record (EHR) adoption and its use have been reported by hospitals globally [1–5], China has promoted nationwide EHR adoption in hospitals for more than a decade [6–10]. Despite the great progress in increasing the adoption rate in recent years, patients’ access to their own EHR data is still not as convenient as imagined. It is universally known that EHR data compose the core of health care big data today [11–19]. Currently, patient engagement is considered a great way to facilitate patient-centred health care services, and patients’ acceptance of their own medical data is the first step, especially through the use of mobile applications, usually smart apps on phones and pads. These trials have been conducted globally, and many countries have cultivated laws, governmental policies, and industrial regulations years ago for legal protection. If EHR data are distributed to patients via smart apps, self-health management, patient satisfaction, and health care quality can be greatly enhanced.

The National Health Commission of China (NHC) has published policies to encourage hospitals and small-sized medical facilities to release EHR data to citizens since 2013 [20–25]. In this paper, we report an investigation with survey and field interviews on the actual adoptions, obstacles and cost savings of EHR data release to patients’ smart apps and conclude with policy suggestions. Our results provide an important baseline for policymakers to craft regulations for patient access to EHR data.

## Materials and methods

The questionnaire adopted a stratified sampling method. According to the report released by the National Center for Health Statistics at the end of February 2020, there are 2762 tertiary hospitals and 9730 secondary hospitals in China, with a ratio of approximately 1:3.5. According to the same proportion of the number of tertiary and secondary hospitals, a sample survey was conducted. Between August 2019 and October 2019, 1350 questionnaires were collected, including 1050 for secondary hospitals and 300 for tertiary hospitals. Among the received questionnaires, 1344 copies were valid, and 6 responses (all are secondary hospitals) had logic contradiction, which released EHR to patients but filled in "None" in the release software. The total effective rate was 99.56%.

The data in this study were from a specific survey, including basic hospital characteristics (hospital size, hospital type, location in which province), four categories of measures to identify to what degree the hospitals allow patients to access their own EHR data, and in what way do the hospitals release EHR data to patients’ smart apps, as detailed below. This survey received responses from 1350 hospitals in China, which covers 30 provinces nationwide. Among these 1344 hospitals, there were 300 tertiary and 1044 secondary hospitals, or 1089 comprehensive and 255 specialized hospitals. The number of hospitals in each province is shown in Table 1.

**Table 1:** Responding hospitals in 30 provinces

Province	Quantity
Anhui	26
Beijing	13
Fujian	22
Gansu	62
Guangdong	148
Guangxi	19
Guizhou	74
Hebei	88
Henan	33
Heilongjiang	41
Hubei	44
Hunan	43
Jilin	1
Jiangsu	57
Jiangxi	33
Liaoning	48
Inner Mongolia	16
Ningxia	19
Qinghai	6
Shandong	63
Shanxi	47
Shaanxi	57
Shanghai	24
Sichuan	96
Tianjin	18
Xizang	9
Xinjiang	5
Yunnan	122
Zhejiang	89
Chongqing	21

## Results

### Hospital survey

The primary of all measures is whether a hospital has already released EHR data to patients or not. According to the responses, 41.37% of hospitals (69% secondary hospitals and 31% tertiary hospitals) have already made EHR data accessible to patients; among them, 37% of secondary hospitals have released EHR, and 58% of tertiary hospitals have released EHR, of which 97.12% are through smart apps and only 2.88% are through traditional format.

Hospitals in China are required to make all of a patient’s medical records available to the patient if requested, according to the *“Regulations on prevention and treatment of medical disputes”* published in 2018. Therefore, the release of medical records in the traditional format, paper copy, is 100% for both outpatients and inpatients.

**Fig. 1:**
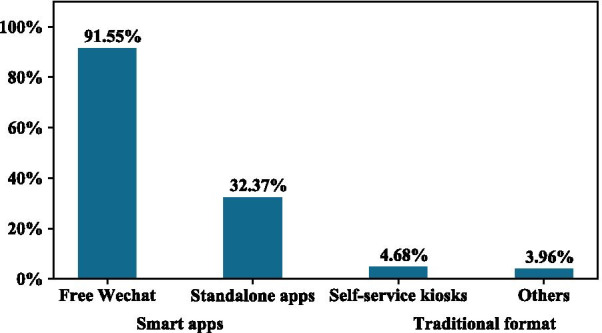
Release software: The release formats of EHR are shown, including traditional formats and smart apps. Free WeChat and standalone apps are smart apps. Self-service kiosks and others are part of the traditional format

Among the smart apps, the most popular is free WeChat, which was used by more than 91% of hospitals to release EHR data. Thirty-two percent of hospitals developed their own standalone apps or used vendors’ apps, as shown in Fig. 1. Self-service kiosks are often seen in hospitals on-site, with volunteers and nurses helping the patients to operate and print out their records. Other approaches, such as mailing and Compact Disc recording, are also sometimes used.

Hospitals release EHR data of inpatients, outpatients, or both. According to the survey, 42.96% of responding hospitals released EHR data to outpatients only, 2.41% to inpatients only, and most hospitals released data to both outpatients and inpatients.

**Fig. 2:**
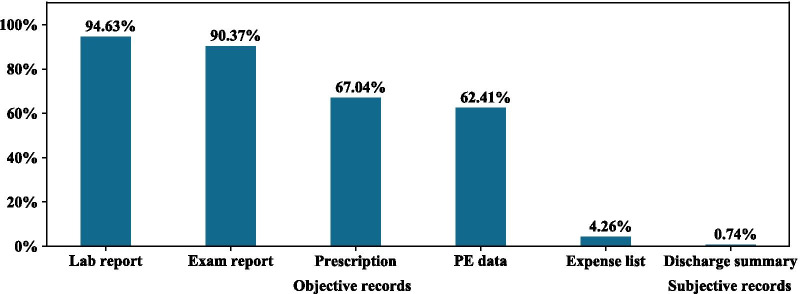
Release content: Release content includes objective content and subjective content. Compared with the objective content, the subjective content is less released

EHR data can be classified into two categories, objective and subjective. Objective data are those that could be generated directly by medical devices or reagents, which include lab reports, examination reports, prescriptions, and images; subjective data are those that are created by medical staff based on their professional knowledge or clinical experiences, which include discharge summary and diagnosis. Among all 1344 responding hospitals, only four hospitals released both objective and subjective EHR data to patients. The top four types of released EHR data (above 60%) are lab reports, exam reports, prescriptions and PE data, which are all in the objective category, as shown in Fig. 2. Release times range from real-time to a delay of a few hours or days, according to hospitals’ specific policies. Most hospitals validate data accuracy before releasing the information to patients.

## Field interview

In addition, we field-interviewed 7 comprehensive tertiary hospitals, which were included in all 1344 responding hospitals, as supplementary data to the survey. Questions included: What is the doctors’ reaction to patients’ access to their EHR data? Does that truly help them improve quality or efficiency? Do patients feel more satisfied after hospitals do so? What is the cost-effectiveness for both hospitals and patients? Among those questions, cost-effectiveness was the chief concern for both the hospital and the patients.

For these 7 hospitals, we interviewed both CIOs and CEOs and collected their IT investment and cost savings via smart apps (for confidential purposes, we cannot list detailed data for each hospital). Then, we made a rough estimation of a normal-sized comprehensive tertiary hospital, with 2 million outpatient services, 80,000 inpatients and 3 billion RMB in revenue per year.

Hospital costs for EHR data release to patients via smart apps consist of multiple components, including IT construction & operation maintenance, cloud storage, web security & E-signature, EHR paper and image materials purchasing and printing. In spite of that, for the match of smart apps, hospitals have to invest more in system development, web infrastructure, security protection, and e-signature. The returns (or the cost savings) of EHR data release are from both sides, the hospitals and the patients.

Table 2 shows that if a normal-sized comprehensive hospital releases EHR data to patients via smart apps (i.e., goes paperless), it can potentially save the hospital 11.8 million RMB per year and save the patients 9.4 million RMB altogether. Although hospitals have to invest in IT construction and operation maintenance, web security, cloud storage, and purchase printing materials, altogether, one comprehensive tertiary hospital can save 18.82 million RMB on both sides per year through this move. However, this estimation is not suitable for secondary and below hospitals.

**Table 2:** Cost savings (million RMB)

Items	IT investment	Cost saving	
	Hospital Cost/year	Hospital/year	Patient/year
IT construction & operation maintenance	1.1	–	–
Cloud storage	0.93	–	–
Web security & E-signature	0.35	–	–
EHR paper & printing	–	4.2	1.1
Images & printing	–	7.6	8.3
Total	2.38	11.8	9.4

## Discussion

Through survey and field interviews, we can see many advantages of releasing EHR data to patients’ smart apps, but there are also some obstacles that need long-term solutions. For example, the primary concern of hospitals is potential legal liabilities and conflicts since physicians’ ‘irregular’ behaviours always lead to poor EHR data quality and may prevent hospitals from releasing those EHR data to patients. There are two possible causes why EHR data quality is not as good as expected. One is that physicians’ documentation might be delayed, may use irregular wording or may be easily copied and pasted, which could become a huge threat to hospitals’ willingness for EHR data release. In addition, doctors always need to make necessary adjustments and modifications to EHR content according to a patient’s disease progression and condition, which could easily be misunderstood as errors and drawbacks by the patients, further arousing medical disputes or even doctors’ defensive EHR writing.

Furthermore, patients’ or their family members’ willingness to know the reality of their health conditions is variable, especially for those who are diagnosed with sensitive diseases, such as infectious disease or cancer. Patients’ family members may choose to tell white lies in consideration of their mental stress. Therefore, smart and smooth EHR data release could become a negative option or an honest mistake.

Third, hospital IT infrastructures and application situations are different from hospital to hospital, and many of the systems inside hospitals are silos, which may narrow down the data type and content; thus, the patients cannot obtain enough data that are needed for future use.

## Conclusions

Leveraging data from a large survey and field interviews, we found that EHR data release in tertiary hospitals is better than that in secondary hospitals. More than half of the comprehensive tertiary hospitals and some of the secondary hospitals have already released EHR data to patients’ smart apps, especially for outpatients and on objective content of EHR data. This act is believed to bring cost savings and efficiency gains to both the patients and the hospitals but is still considered spontaneous, without legal support and government regulation. The NHC is currently giving this issue much attention for the protection of patients’ information security and may issue suitable policies for future regulation and application.

## Data Availability

The datasets generated and/or analysed during the current study are not publicly available because our data belong to the National Health Commission, and they do not agree to make it public.

## Abbreviations

NHC: National Health Commission of the People’s Republic of China
EHR: Electronic health records
APP: Application
IT: Information Technology
PE: Physical Examination
CEO: Chief Executive Officer
CIO: Chief Information Officer
